# The effect of different workplace nanoparticles on the immune systems of employees

**DOI:** 10.1007/s11051-017-4004-6

**Published:** 2017-09-13

**Authors:** Natalja Kurjane, Tija Zvagule, Jelena Reste, Zanna Martinsone, Ilona Pavlovska, Inese Martinsone, Ivars Vanadzins

**Affiliations:** 1 Institute for Occupational Safety and Environmental Health, Riga Stradins University, Dzirciema Street 16, Riga, LV-1083 Latvia; 2 Faculty of Material Science and Applied Chemistry, Institute of Silicate Materials, Riga Technical University, Paula Valdena Street 3/7, Riga, LV-1048 Latvia

**Keywords:** Wood and metal nanoparticles, Immune system, Cytokines, Adhesive molecule, Nasal lavage, Environmental, Health and safety effects

## Abstract

Currently, nanoparticles are widely present in the environment and are being used in various industrial technologies. Nanoparticles affect immune functions, causing different immune responses. The aim of the current study was to evaluate several cytokines, interleukin (IL)-1b, IL-6, IL-8, tumour necrosis factor-a (TNF-α), interferon-γ, adhesive molecule sICAM-1, macrophage inhibitory protein 1a (MIP1a) and secretory immunoglobulin A, in nasal lavage fluid and in the peripheral blood of healthy subjects exposed to workplace nanoparticles. Thirty-six employees from three different environments were examined: 12 from a metalworking company, 12 from a woodworking company and 12 office workers. The nanoparticles in the different workplaces were detected in the air in the immediate vicinity of the employees. The particle number concentration and surface area values were significantly higher in the workplaces of the metal- and woodworking industries, but concentrations of mass were lower (the measurements were performed by an electrical low-pressure impactor ELPI+). Energy dispersive X-ray spectroscopy (EDS, an attachment to a high-resolution SEM) was used to provide elemental analysis or chemical characterization of the dust particles in a low-vacuum field-free mode operating at a potential of 15 kV spot 3.0. The technique used provided quantitative and spatial analyses of the distribution of elements through mapping (two to three parallel measurements) and point analysis (four to five parallel measurements). Samples from the metal industry contained more ultramicroscopic and nanometric particles, e.g. toxic metals such as Zn, Mn and Cr, and fewer microscopic dust particles. The nasal lavage and peripheral blood were taken at the beginning and the end of the working week, when immune indices were measured. Our data showed a statistically significant increased level of the pro-inflammatory cytokine TNF-α in serum in both exposed groups compared with office workers as well as a higher level of TNF-α in workers from the woodworking company compared with the metalworking employees. We found an elevated level of IL-6 in the exposed groups as well as an elevated level of IL-8 in the nasal lavage in woodworking employees after work.

## Introduction

Currently, people are affected by different types of nanoparticles everywhere, mostly via exposure caused by human activity. Nanoparticles are defined as primary particles with at least one dimension < 100 nm (Warheit 2004). Nanoparticles have useful actions (for example, in medicine and cosmetology) but are also harmful for biological systems. The workplace is usually a major source of nanoparticles that influence different biological systems of the body and, as a result, may cause a disease. The pathogenetic mechanisms of its action are still unclear despite the large number of investigations throughout the world. The nanoparticles in the workplace can penetrate to the tissues with different ways, for example, through the skin, via the digestive tract or through the nasal olfactory epithelium, causing damage to the central nervous system (Larese et al. 2009; Borm et al. 2006). Nasal mucosa is the first part of the respiratory system to be exposed to different airborne environment pollutants found in the workplace (Horvath et al. 2011). Mucosa phagocytic cells, as a part of innate immunity, represent the first line of protection from nanoparticles (Boraschi et al. 2012; Karavitis and Kovacs 2011; Boraschi and Duschl 2014; Song et al. 2014). Innate and adaptive immune systems participate in this protection.

Nanoparticle action is influenced by a number of parameters, including particle type, airborne concentration, size distribution, water solubility, chemical reactivity, frequency and duration of exposure, interactions with other chemicals and the individual’s immunological condition (Winder 2004; Petrarca et al. 2015; Koponen et al. 2011; Saptarshi et al. 2013; Gamucci et al. 2014). A large surface area of inhaled nanoparticles is sufficient to initiate inflammation (Borm et al. 2006; Bakand et al. 2012). Nanoparticles can cross cell membranes and enter into tissue cells (Peters et al. 2004). The small size of nanomaterials may also induce a direct cause of cellular injury due to particle-cell interactions. Biological effects of nanoparticles are different, such as the formation of reactive oxygen species (ROS) generation, oxidative stress, mitochondrial perturbation, inflammation, uptake through reticulo-endothelial system, protein denaturation, phagocytosis impairment, endothelial dysfunction, generation of neoantigens, altered cell cycle regulation and DNA damage (Nel et al. 2006; Karlsson et al. 2008; Jang et al. 2010). Nanoparticles at cytotoxic doses can cause necrosis or apoptosis of the immune cells, but at non-cytotoxic doses, they provoke pro-inflammatory effect by the activation of the certain inflammatory cytokines (Petrarca et al. 2015).

The goal of the current work was to evaluate and compare the influence of different nanoparticles (encountered in woodworking, metal processing and office environments) on the immune system, both local (in the mucosa) and systemic (in the peripheral blood), with the suggestion that the effect of the workplace nanoparticles can cause inflammatory reactions in the body and could cause a predisposition for the development of possible inflammation-associated diseases in the future.

## Materials and methods

### Study design

The current study was designed to evaluate several immunological tests in the blood serum and nasal lavage of office, wood and metal processing workers before work on Monday at the beginning of the week and at the end of the working week on Thursday afternoon. The nanoparticles in the different workplaces were detected in the air in the immediate vicinity of the employees. The results were compared among all examined groups. The main task of the study was evaluating the occupational exposure of nanoparticles with respect to increasing the concentration of pro-inflammatory cytokines among healthy subjects from different occupational environments.

### Ethics

The study was approved by the ethics committee of Riga Stradins University. All study participants received clear verbal and written descriptions of the aims and the course of the procedure and signed an agreement to participate in the current study.

### Sample characteristics

Thirty-six employees (in the age range of 26 to 70 years) were included in the current study, which occurred in January–February 2015. Twelve persons were from a metalworking company, 12 from a woodworking company and 12 were office workers (the control group). The selection of these groups was made because wood and metal are common industries in Latvia. A total of 42% of the metalworkers smoked, 25% of the woodworkers smoked and 17% of the office workers smoked. Regarding gender, 58% of the workers in the metalworking group were women, compared to 42% in woodworking and 33% in the office group. Health conditions were evaluated by a certified physician and otolaryngologist. Only 8% of the employees had high blood pressure in the metalworking and office group, while in the woodworking group, this was the case for 50% of employees. The metalworkers and office employees did not have any upper airways diseases, but 8% of woodworking employees had chronic bronchitis. A total of 17% of metal and woodworking employees had allergic anamnesis, as opposed to 42% of the office workers. In the current examination, none of the selected subjects had acute symptoms of upper respiratory tract infections. No subjects were taking anti-inflammatory medications.

The nasal lavage and peripheral blood, in which immune indices were measured, were collected at the beginning and at the end of the working week—a difference of 4 days. Nasal lavage samples were collected on Monday in the morning and Thursday in the afternoon. Nasal lavage samples were taken by 10 mL saline injected manually with a 20-mL syringe into the nasal cavity through one nostril and collected from the second nostril. The same procedure was used to collect nasal lavage of the other part of the nasal cavity. Nasal lavage samples from both nostrils/both parts of the nasal cavity were collected in sterile containers. Nasal lavage samples immediately were transported to the laboratory, stored in the cold and analysed. Blood samples were obtained from the cubital vein in 5-mL sterile vacutainers coated with EDTA (ethylenediaminetetraacetic acid) for the cytokine detection and without EDTA for the immunoglobulin detection. Vacutainers were labelled and immediately transported to the laboratory within 2 h.

### Equipment and procedure

The immunological measurements were made by enzyme-limited immunosorbent assay (ELISA) procedures in blood serum and nasal lavage using standard kits: for the measurement of tumour necrosis factor-a (TNF-α), interleukin (IL)-1β, interferon-γ (IFN-γ), IL-8, sICAM and MIP-1α—from Invitrogen Corporation (USA); IL-6—by Millipore (USA); C—Biorbyt (UK) and secretory IgA—Demeditec Diagnostics GmbH (Germany). All cytokines and adhesive molecules were measured by commercially available ELISA kits according to the instructions of the manufacturer. All samples were tested in duplicate, and the mean of the two readings was taken.

In our study, the investigated levels of IL-8 in the nose lavage had the limitations of the methods used; some of the results we obtained were just above the level of 25 mg/L without a clearly shown exact number; therefore, we decided to estimate the data using “below and above” relative to a certain limit.

The nanoparticles in the nanoscale range < 100 nm are described across the three different environments: in the office and in the welding (metalworking) and woodworking industries. The specific personal sampling equipment for total dust concentration analyses was used for the measurement (Millipore nitrocellulose membrane filters 0.025 μm VSWP; personal sampling pumps: Gilian 3500). The concentrations of the dust particles (including nanoparticles) were detected by an electrical low-pressure impactor (ELPI+, Dekati Ltd., Finland), where the 14-stage cascade impactor distributed the number of particles and surface area concentrations by particle size. Particle distribution was detected in 14 stages with the following cut-off points (geometric mean diameter (aerodynamic) μm): 1st cut-off point—0.01, 2nd—0.02, 3rd—0.04, 4th—0.07, 5th—0.09, 6th—0.16, 7th—0.32, 8th—0.49, 9th—0.79, 10th—1.23, 11th—1.96, 12th—3.09, 13th—5.17 and 14th—8.15.

The surroundings of all three workplaces were similar; they were located in a city but away from the main streets and major roads. All workplaces were equipped with ventilation systems and, in the cases of the metal and wood industries, also with local exhaust ventilation. Real-time measurement data from one working shift (8 h including breaks) were collected by the electrical low-pressure impactor (ELPI+, Dekati Ltd., Finland). Measurements were performed approximately 1.5 m from the floor and as close to the worker as technically possible (1–2 m from the operator, as the ELPI+ measuring device has limited closer access to the breathing zone of the operator because the instrument is not intended for personal sampling). During measurements, all workers performed their main daily tasks: for office workers, working at the computer (90% of an 8 h shift) and communicating with clients and colleagues (10% of an 8 h shift); for welders, welding (80% of an 8 h shift), grinding material for welding (5% of an 8 h shift) and preparing raw material for welding or removing produced material from the welding table (15% of an 8 h shift); and for woodworkers, polishing materials (80% of an 8 h shift). Therefore, particle sampling data includes typical occupational exposure caused by welding and polishing processes.

The data were saved every second. All measurements and calculations of particles were done according to international standards and methods (LVS EN ISO 10882-1:2002 and NIOSH MAM Method 0500). The measurements for workplace particles across the three different environments described above were also analysed by SEM (NovaNanoSEM 650). Energy dispersive X-ray spectroscopy (EDS, an attachment to a high-resolution SEM) was used to provide the elemental analysis or chemical characterization of the dust particles in a low-vacuum field-free mode operating at a potential of 15 kV spot 3.0. The technique provided quantitative and spatial analyses of the distribution of chemical elements through mapping (two to three parallel measurements) and point analysis (four to five parallel measurements). Samples were taken by ELPI+ on Al foils and were analysed by SEM EDS in order to describe the chemical content of particles of different stages. For visual comparison, particles were viewed with a Leica M420 stereomicroscope (paired with a Leica DC camera).

All data was transported to Microsoft Excel and the statistical programme IBM SPSS Statistics version 20 where the statistical analysis was conducted. The significance level was set at 0.05. Appropriate statistical methods were employed according to the shape of the data distribution. Taking into account the small number of study participants, the distribution of the data was not normal. So, for the comparison of groups to each other, non-parametrical tests (e.g. the chi-square test and the Mann-Whitney test) were used. For achieving a precise work environment effect evaluation, data were weighted by smoking status, alcohol consumption and health-related information (data received from a questionnaire) before analysis using an integrated weighting approach.

## Results

The nanoparticles were measured in three work spaces simultaneously with the collection of blood and nose lavage samples from the employees.

The results of particle number and surface area concentrations showed the highest concentrations in the metal industry (see Table 1).

**Table 1:** The results of particle number and surface area concentrations

Industry/workplaces	Parameter	Concentration of nanoscale particles (range 6–100 nm)	Concentrations of total particles (6 nm–10 μm)	Count median diameter of particles (μm)
Office environment (background level)	Number concentration, 1/cm^3^	10,835	11,707	0.039
	Surface area concentration, μm^2^/cm^3^	83	513.6	0.641
Metal industry (welding and grinding)	Number concentration, 1/cm^3^	216,153	272,370	0.054
	Surface area concentration, μm^2^/cm^3^	3382	21,644.9	0.463
Woodworking industry (grinding and polishing)	Number concentration, 1/cm^3^	80,120	80,510	0.012
	Surface area concentration, μm^2^/cm^3^	88	666.2	2.042

**Fig. 1:**
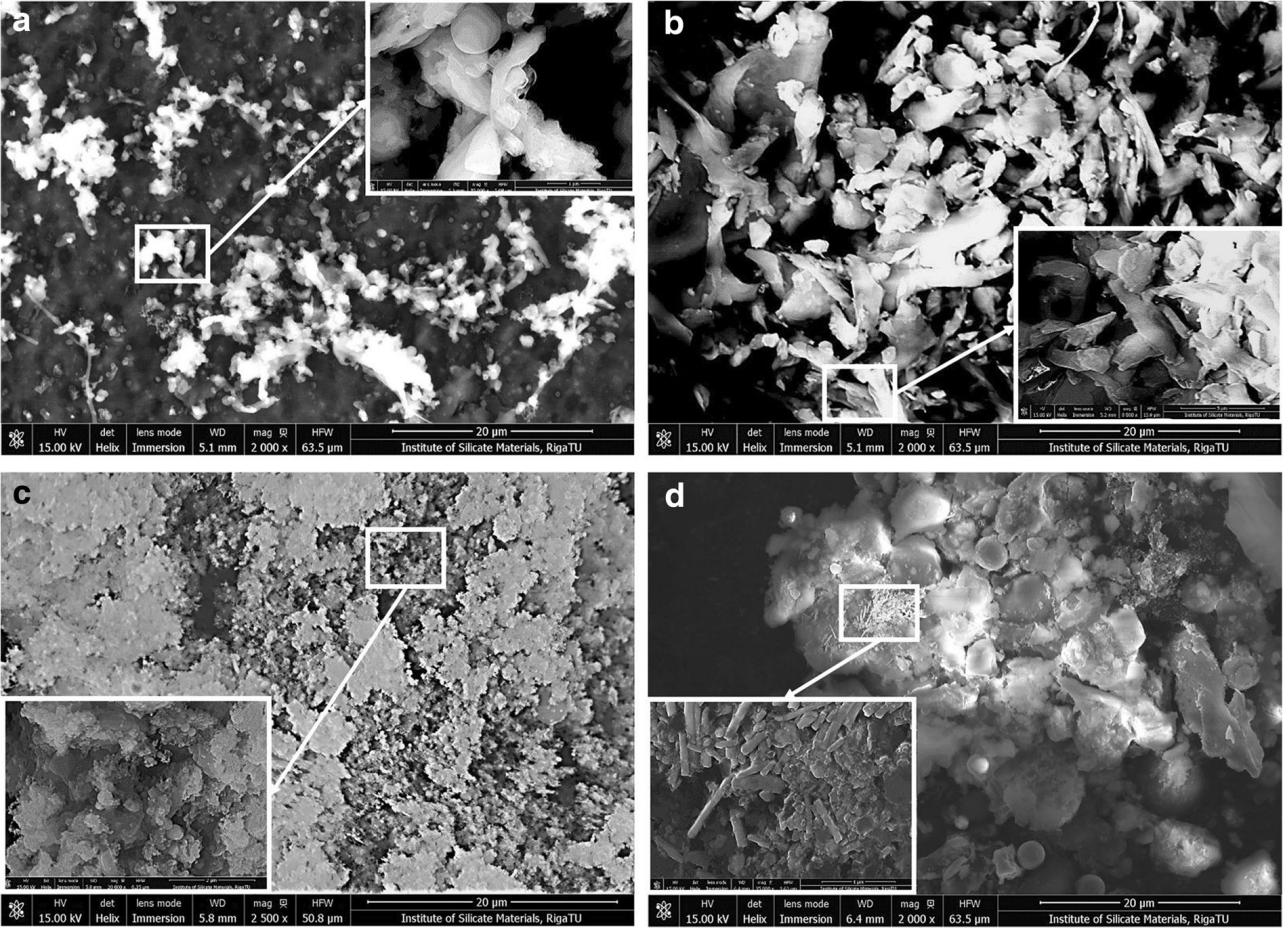
SEM images for dust samples from the wood industry at stages 8 (**a**) and 14 (**b**) and the metal industry at stages 8 (**c**) and 14 (**d**) in field-free and immersion modes (due to the different sample natures). Bar 20 = μm

The measurements for workplace particles across the three different environments described above were also analysed by SEM (NovaNanoSEM 650). It was confirmed that all of the main inorganic elements constituting the particles are present; for example, sodium, calcium, silicon, iron, magnesium, manganese, zinc and chromium as well as chlorine, potassium and sulphur were observed. Fractions from the metalworking samples visually consisted commonly of dark brownish glossy particles at all stages. Otherwise, the fractions from the woodworking industry samples were greyish below stage 9 and had different sizes of fibres up to level 9; this was confirmed later by SEM EDS (Fig. 1).

Samples taken by ELPI+ on Al foils were analysed by SEM EDS in order to describe the chemical content of particles at different stages (Fig. 1). The average results and mean arithmetic deviations from two to three parallel mappings and four to five parallel point results are shown in Table 1. The particles at stage 2 to stage 8 were fixed on carbon PELCO tabs using Al foils, but those at stage 9 to stage 15 were collected and fixed on carbon tabs directly (without Al foils) due to their lightweight characteristics and instability. Poorly fixed dust particles were eliminated with compressed air.

Some differences were detected in particle chemical compositions depending on the industry. Particles from the metalworking industry comprised Fe > C > Si > Ca > Zn > Cr > Mn (in decreasing order), while particles from the wood processing industry comprised C > Si > Ca > Fe > Mo > Na. The predominant elements were carbon and oxygen, and there were also traces of chlorine and sulphur that possibly originated from organic compounds or from inorganic oxides, acids and salts (Pavlovska et al. 2016).

The main source of particle pollution in office environments is the ventilation systems (ambient air), but there are also printing/copying processes and the additional source of carpets on the floors. In the case of metal processing, the main sources are welding and grinding processes. In the case of wood processes, particles are produced during polishing processes.

**Fig. 2:**
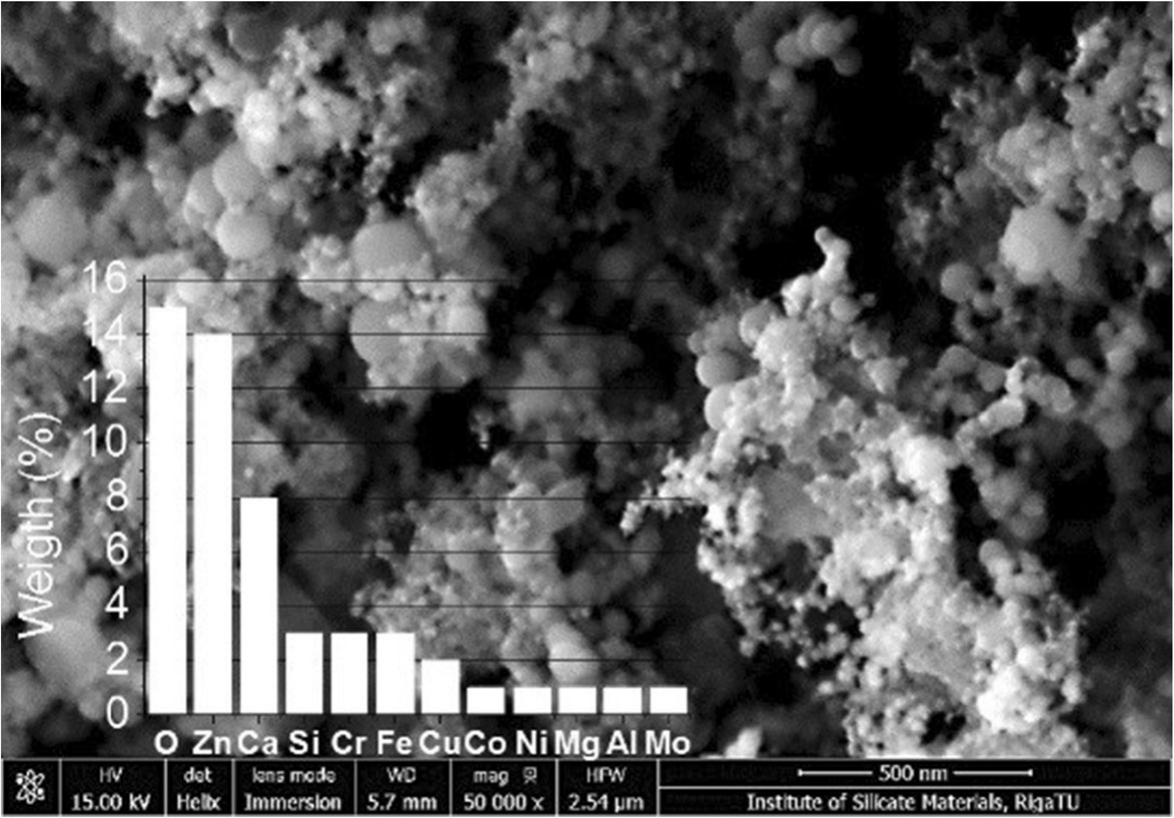
Elemental analysis of particles in metal industry air dust at stage 6 (spherical silica particles with different dimensions, < 50, 50–130 and 130–370 nm; Fe_2_O_3_ and ZnO nanoparticles < 30 nm; elemental overlay (wt%): O—15; Zn—14; Ca—8, Si, Cr and Fe—3 each; Cu—2 and Co, Ni, Mg, Al, Mo and Ag—1 each). Bar = 500 nm

Particle distributions were collected by number and mass concentrations in all three environments. According to the mass concentration (mg/m^3^), the highest concentrations were estimated in the following cut-off points (descending order): in offices, from 12th–14th; in metal processing, 14th, 13th, 12th, 8th and 7th; and in wood processing, from 12th–14th. According to the particle number concentration (particles/cm^3^), the highest concentrations were estimated in following cut-off points: in offices, 2nd, 3rd and 4th; in metal- processing, 1st, 4th, 5th and 6th; and in wood processing, 1st, 2nd, 3rd, 4th and 5th. The results showed that fine and ultrafine (nanoparticle) size particles could directly influence workers’ health because particle number concentrations in these cut-off points were very high (Fig. 2).

**Fig. 3:**
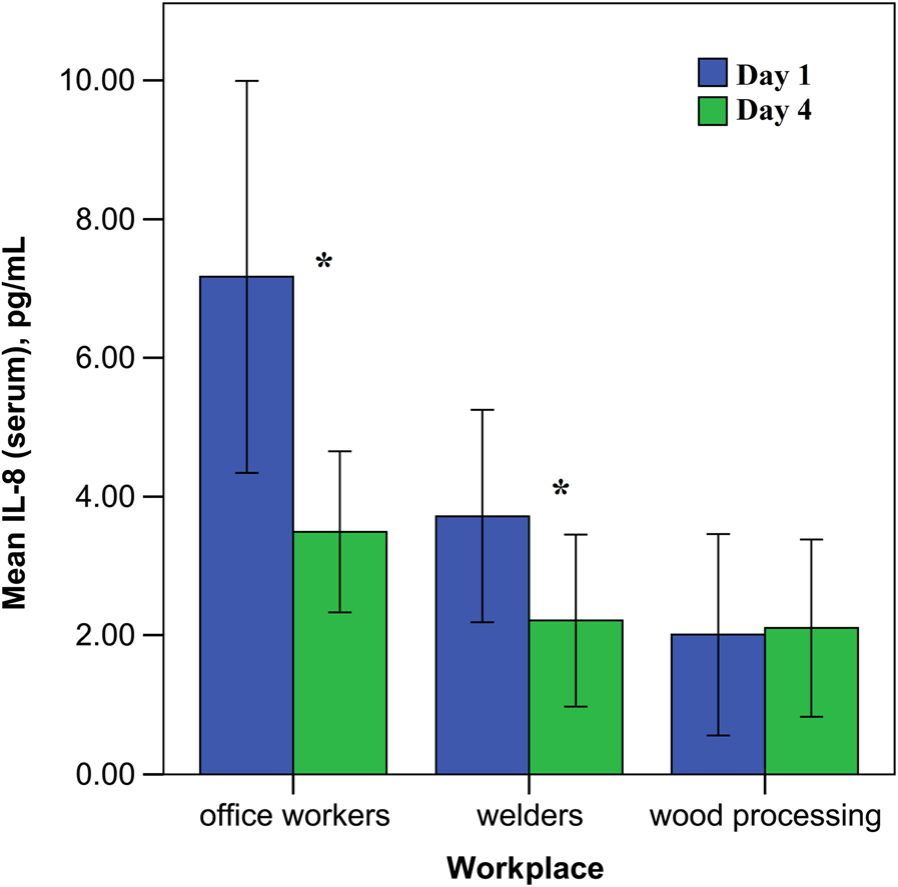
The serum level of interleukin-8 (IL-8) among the examined groups at the beginning and the end of the working week (error bars indicate 95% confidence interval, asterisks—*p* < 0.05)

Simultaneously, with the measurements of nanoparticles from the different workplaces, the immunological tests were performed in the peripheral blood and nasal lavage. Immunological data shows that, in office workers, the concentration of IL-8 in blood serum was significantly (*p* < 0.05) elevated at the beginning of the work week and then decreased within 4 days during the week (see Fig. 3). Secretory IgA in the nasal lavage fluid, conversely, was higher at the end of the work, but not significantly. In the other examined immune parameters, office workers did not undergo any changes at the beginning and at the end of the working week.

The level of IL-8 in the nose lavage was analysed depending on if the percent was below or above 25 mg/L (due the test system limitation). For the evaluation of the inflammatory reactions in the nasal mucosa, it is important that an elevated level was observed only for IL-8; therefore, we estimated the data to be above 25 mg/L.

**Fig. 4:**
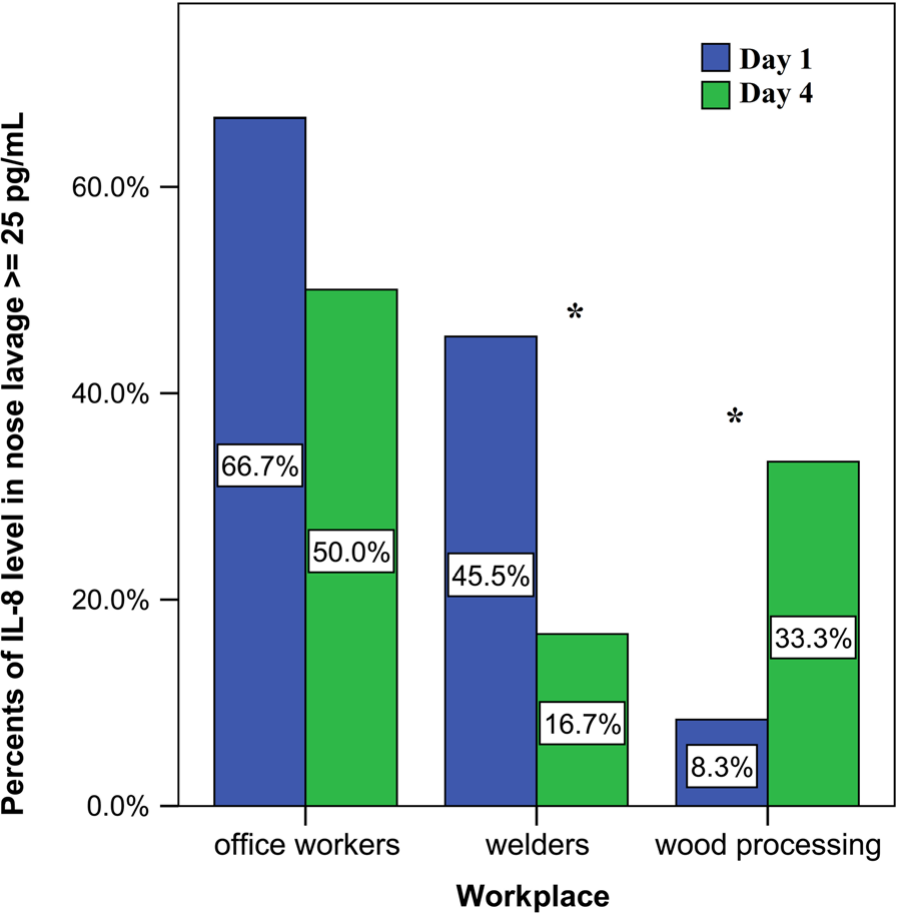
The percentage of interleukin-8 (IL-8) concentration above 25 pg/mL in the nose lavage among examined groups at the beginning and the end of the working week (asterisks indicate *p* < 0.05)

In office workers, IL-8 was elevated at the beginning of the work week and then decreased at the end of the week. In metalworking employees (welders), the concentration of IL-8 was lower than in office workers, but higher than in woodworkers on Monday and at the end of the work much lower than in office workers. In woodworking employees, the concentration of IL-8 in the nose lavage was decreased at the beginning of the work week, but increased at the end of the working week (see Fig. 4).

Secretory IgA levels and IL-6 concentrations among metalworking employees after work at the end of the week in the nasal lavage were elevated, but not significantly (*p* > 0.05).

**Fig. 5:**
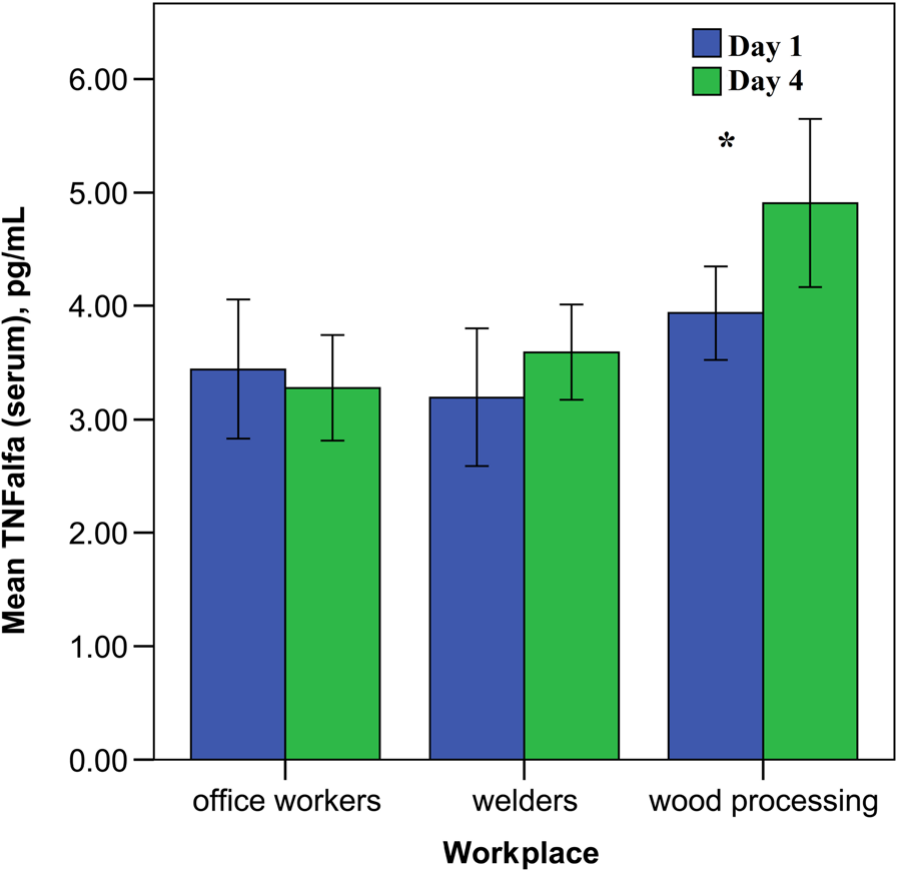
The level of serum TNF-α in the investigated groups depending on the work day (error bars indicate 95% confidence interval, asterisks—*p* < 0.05)

Our data showed a statistically feasibly increased level of pro-inflammatory cytokine TNF-α in the blood serum of the woodworking employees after work (*p* > 0.05) (see Fig. 5). Interferon-γ and secretory immunoglobulin A (sIgA) level was elevated in the nasal lavage fluid at end of the work week, but not to a significant degree.

Comparing all the immune parameters among the investigated employees, it was found that IL-8 in serum was statistically higher at the beginning of the working week among office workers than among metalworking employees. However, at the end of the working week, the results became similar. In woodworking employees’ nasal lavage, the level of IL-8 at the end of the working week increased.

Interleukin-6 and TNF-α, in contrast, did not differ between the two groups, office workers and metalworkers, before work at the beginning of the week. However, at the end of the working week, IL-6 and TNF-α were significantly higher (*p* < 0.05) among metalworking employees in blood serum as well as in nasal lavage fluid.

The concentration of adhesive molecule sICAM-1 and macrophage inhibitory protein 1a (MIP1a) as well as interferon-γ (IFN-γ) did not differ significantly among all investigated groups (both in the nose lavage and in the serum).

**Fig. 6:**
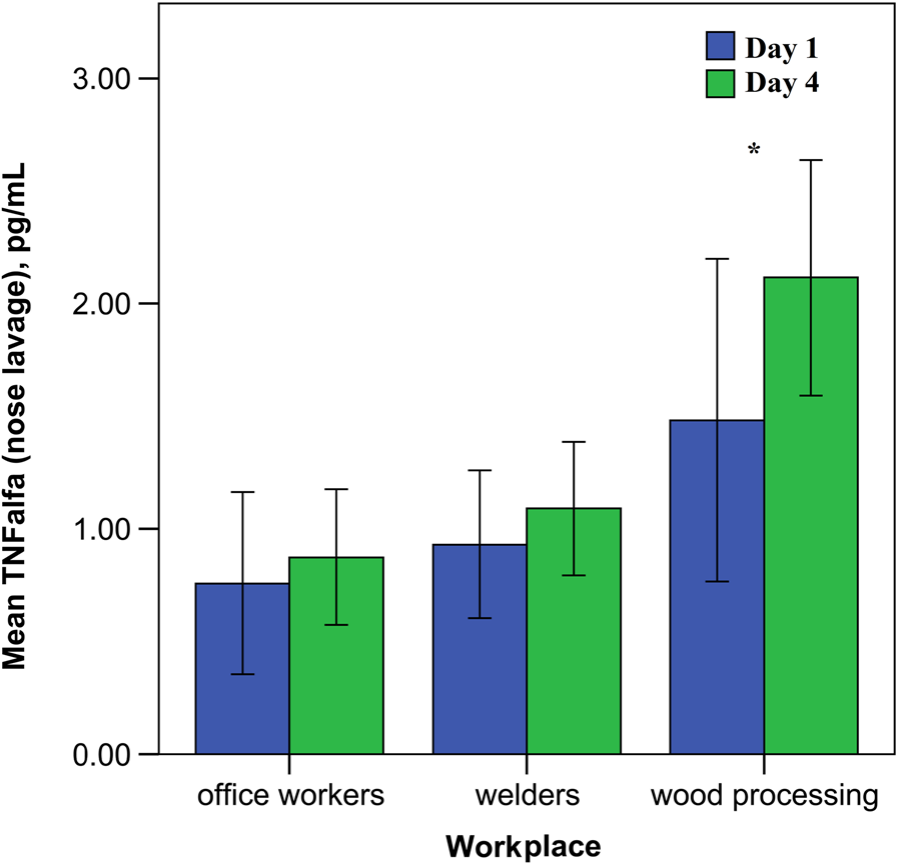
The concentration of TNF-α in the nose lavage of the investigated groups depending on the work day (error bars indicate 95% confidence interval, asterisks—*p* < 0.05)

Our data showed that the level of pro-inflammatory cytokine TNF-α was statistically higher in the serum and in the nasal lavage fluid (see Fig. 6) among workers from the woodworking company compared to metalworking employees. The conducted study showed the woodworking employee group was more exposed to the working dust particles in comparison with the other investigated groups.

We also compared smokers and non-smokers in all investigated groups (see Table 2). Statistically significant differences were not observed between smokers and non-smokers in most of the cytokine levels in all study groups and on different days, except TNF-α in the nose lavage in welders and wood processing workers, as well as IgA in the serum in office workers on the first day of the study and IL-8 in the nose lavage in welders on the fourth day of the study. Additionally, we examined the alcohol consumption, but we did not observe significant differences between the groups depending on this.

**Table 2:** Median values of selected cytokines by working day, workplace and smoking status (asterisks indicate *p* < 0.05 for comparison of parameters between smokers and non-smokers)

Cytokines	Day 1						Day 4					
	Office workers		Welders		Wood processing		Office workers		Welders		Wood processing	
	Smoker	Non-smoker	Smoker	Non-smoker	Smoker	Non-smoker	Smoker	Non-smoker	Smoker	Non-smoker	Smoker	Non-smoker
sICAM (serum), ng/mL	259.50	285.50	273.00	275.50	201.00	241.00	319.00	264.00	229.00	257.00	215.00	245.00
sICAM (nose lavage), ng/mL	0.36	0.94	0.90	0.94	2.32	1.07	0.49	1.26	0.84	1.76	2.33	1.11
IL-6 (serum), pg/mL	0.39	1.31	2.25	1.71	2.12	1.44	0.46	0.77	1.98	1.58	0.77	1.85
IL-6 (nose lavage), pg/mL	0.66	#	#	0.25	3.50	1.04	#	#	1.99	1.99	6.11	4.39
IL-8 (serum), pg/mL	12.93	5.47	2.81	3.39	1.03	0.94	2.14	3.08	1.57	2.25	1.10	1.34
IL-8 (nose lavage), pg/mL	25.00	25.00	25.00	22.79	22.84	22.30	22.85	25.00	22.33*	22.57*	22.65	23.00
TNF-α (serum), pg/mL	3.41	3.48	3.30	3.09	3.51	4.37	3.25	3.24	3.71	3.50	6.23	5.10
TNF-α (nose lavage), pg/mL	0.82	0.56	0.43*	1.24*	0.44*	1.37*	0.44	0.85	1.30	0.95	2.11	2.57
IL-1b (nose lavage), pg/mL	1.66	1.36	1.07	1.17	0.87	2.00	1.06	1.35	0.81	0.90	4.61	2.08
IFN-γ (nose lavage), pg/mL	#	#	6.83	8.10	15.90	#	3.90	#	17.08	9.95	18.86	12.68
IgA (serum), g/L	3.76*	2.25*	1.32	2.58	2.43	2.11	3.40	2.55	1.45	2.53	1.99	1.79
IgA secr. (nose lavage), m/mL	4.19	1.89	2.03	3.45	10.48	1.46	5.20	2.48	2.96	3.40	22.66	5.81

^#^Not applicable

## Discussion

The number of inflammatory diseases is increasing. The reasons are different; people face various degrees of exposure to nanoparticles, natural and engineered, and to biological endotoxins. Statistically, in Latvia, the number of occupational diseases is increasing, maybe because better diagnostics and consultations have become available in recent years. However, in Western European countries, the number of these diseases has decreased.

Wood processing is one of main industrial branches in Latvia. Furthermore, there is a chance (because of a lack of appropriate ventilation systems, lack of workers’ safety behaviour, etc.) that nanoparticles and particles less than 1 μm have been produced during wood polishing and grinding processes. It is very important to note what kind of exposure we describe to compare the potential impact of particle exposure on workers’ health. For example, in the case of particle mass concentration, it is clear that exposure is very high in the range of inhalable particles. However, in the case of particle number concentration, it is very high in range of respirable, fine or ultrafine particles (Pavlovska et al. 2016). Currently, total dust, inhalable and respirable dust mass concentrations are used to describe the whole impact to workers’ health, but there are many discussions on particle number concentration as a better parameter for the characterization of workers’ health effects. According to the results of occupational environment measurements, workers have been sent to occupational physicians to undergo medical check-ups considering occupational risk factors (particle exposure). There is a possibility that in acceptable total dust mass concentrations, there are very high concentrations of fine and ultrafine particles which create adverse effects on workers’ health (respiratory, cardio-vascular, etc.); immunological analyses could be the first step of the evaluation of these effects. We identify these three occupational environments to characterize particle concentration levels (also distribution) and to identify potential health effects according to immunological parameter results.

In general, nanoparticles, as well as other occupational or non-occupational particles, can cause similar immune responses through the activation of innate or adaptive immunity. The upper respiratory tract is the first assembly point on the way of these inhaled particles. Of course, the coarse particles cause stronger immune response than small ones. The feasibility of differentiating the immune response to coarse particles from the response to nanoparticles is nearly impossible. Of course, organic and inorganic dust may influence the results differently, but it was not our aim in this study to evaluate the chemical composition of the particles. Most investigations confirm that the permanent occupational influence of inhaled nanoparticles can cause inflammatory upper airway diseases by promoting increased susceptibility to infectious and allergic diseases (Hox et al. 2014; Kononenko et al. 2015; Zolnik et al. 2010).

One of the biomarkers of immune dysregulation leading to inflammatory processes is the level of pro-inflammation cytokines (Kononenko et al. 2015; Boraschi and Duschl 2014; Elsabahy and Wooley 2013). TNF-α, IL-1β and IL-6 are pro-inflammation cytokines released by macrophages and dendritic cells. In the current study, we focused our investigations on how the work-related nanoparticles locally influence the immune response in the nasal mucosa and systemically in the peripheral blood serum. Several cytokines, as well as adhesive molecules and sIgA, which participate in innate and adaptive immune response, were detected in the blood serum and in the nose lavage.

IL-1 and IL-6 are mainly produced by activated macrophages, endothelial cells and various other cell types (Boraschi et al. 2012; Boraschi and Duschl 2014). These cytokines mediate inflammation by inducing the synthesis of cell adhesion molecules (CAM) within the vascular endothelium. IL-1 binds to receptors on leukocytes and endothelial cells, which activates an inflammatory response. Adhesive interactions between endothelium and circulating cells are crucial for the development of inflammatory reactions. Both IL-1β and IL-6 mediate inflammation by binding to their respective receptors on endothelial cells to induce the expression of leukocyte adhesion molecules such as ICAM-1. ICAM-1 interacts with counter receptors on the surface of leukocytes to mediate the migration of leukocytes, and it is also involved in the arrest of leukocytes on inflamed endothelium (Lesniak et al. 2013; Winter et al. 2002; Celic et al. 2015). The macrophage inhibitory protein 1a (MIP1a) takes part in the leukocyte chemotaxis and correlates with IL-8 (Gamucci et al. 2014). Combined with the immune parameters, this gives the possibility of evaluating the inflammatory response to the work-related nanoparticles.

The data that we reported in this study showed a significantly increased level of TNF-α in the serum and nose lavage in both exposed groups (in woodworking and metalworking employees). Similar data with increased levels of the TNF-α and IL-6 was shown by Sigsgaard et al. (2000) in experimental exposure to organic dust. It is known that IL-8 is a chemokine involved in the recruitment of immune competent cells from the vascular area to the nasal mucosa, which correlates with the adhesive molecule ICAM-1 (Winter et al. 2002; Lozano-Fernandez et al. 2014; Dokic et al. 2011). Peters and co-authors observed the pro-inflammatory effects in human endothelial cells after exposure to cobalt, SiO2 and TiO2 nanoparticles, through enhancing IL-8 cytokine production (Peters et al. 2004). Sigsgaard et al. also found an increase in IL-8 concentration in the nose lavage among exposed workers that can point to the activation of macrophages after engulfing occupational nanoparticle exposure (Sigsgaard et al. 2000; Petrarca et al. 2015).

In our study, we also found a much higher level of IL-8 in woodworking employees in the nasal lavage after work. We suggest that there is a difference between the concentrations and diameters of the measured metal and wood nanoparticles (see Table 1), and this can affect the local immune cells differently.

The macrophage inhibitory protein 1 (MIP1) is a chemokine that regulates the activation and migration of leucocytes to the inflammation site. MIP1 activity as well as ICAMs usually correlate with that of IL-8. Leukocytes after activation can transmigrate into tissue, binding to endothelial cells via ICAM-1 (Kuhn et al. 2014). In the literature, ICAM-1 expression has not been detected for unexposed normal epithelial cells (Lesniak et al. 2013). However, persons with airway infections, allergic rhinitis or bronchial asthma have increased ICAM-1 in blood and in nasal lavage fluid (Winter et al. 2002). An increased level of ICAM could be a part of the inflammatory response in the nasal mucosa. The increased expression of pro-inflammatory cytokines in the nasal lavage (such as TNF-α and IL-1β) among healthy subjects was found after exposure to ozone (Dokic et al. 2011). Pro-inflammation cytokines induce expression of ICAM-1 and selectins, as well as the synthesis of the IL-8 causing their up-regulation (Dokic et al. 2011).

Despite our suggestions, we did not find the differences in the concentrations of the adhesive molecule sICAM-1 and MIP1a among all investigated groups (both in the nose lavage and in the serum). This may be because of the small groups of the investigated employees or insufficient sensitivity of these parameters for the evaluation of the inflammatory reactions.

Additionally, we analysed the role of confounding factors, such as alcohol and cigarette smoke. We compared smokers and non-smokers, as shown in Table 2. No differences were found among all examined groups. We provided a questionnaire for all workers about alcohol consumption and we examined IL-8 depending on this question, but the result was the same, without any significant differences. Perhaps, the answers werе nоt honest; it is difficult to comment, but we do not have any suggestion as to why we had such results. It may be we must continue our work and examine more workers to reach correct conclusions about IL-8. The health conditions of the employеes were evaluated via a questionnaire. There were questions not only about alcohol and cigarette smoke but also about medications, including the kind of medications (the name), dosage and regularity. We excluded persons who had been taking anti-inflammatory medications.

Interferon-γ (IFN-γ) recruits the macrophages to the presenting antigen site (Elsabahy and Wooley 2013). Some information was found in the literature about the investigation of IFN-γ in persons exposed to different types of nanoparticles. For instance, Hanley et al. found that ZnO nanoparticles increased the expression of IFN-γ, IL-12 and TNF-α (Hanley et al. 2009). Petrarca et al. (2006) determined that cobalt nanoparticles induced enhanced IFN-γ and TNF-α, similar to auto-immune and allergic contact dermatitis responses. However, in our study, IFN-γ was not affected in the investigated groups. Moreover, we did not find any changes in IL-1β, IL-6, ICAM-1 or MIP1 concentrations during the working week, despite our finding TNF-α and cytokine, which also reflects inflammation. It was difficult to explain the lack of changes in production of several of the aforementioned cytokines, excluding the significantly elevated TNF-α. Of course, this could be due to the small size of the group of workers that were investigated; despite that, the tendency of certain differences is shown. A few studies regarding the effects of wood or metal nanoparticles on subjects in the workplace have been reported (via PubMed).

The nasal mucosa also contains B cells, which provide adaptive immunity (Kirkeby et al. 2000. Activated B cells produce sIgA, which works as the effector mechanism of the mucosal immune system (Kirkeby et al. 2000; Holmgren and Czerkinsky 2005). The nanoparticles, as airborne substances, are recognized by immune cells as “invaders” with ensuing destruction (Luo et al. 2015). Secretory immunoglobulin A is independent of blood immunoglobulin A and defends the mucosa (Kirkeby et al. 2000). This antibody plays a critical role in mucosal immunity. We expected that sIgA could be elevated after exposure to the working nanoparticles in our study due to possible local inflammation or irritation of mucosa, but we did not find any significant changes.

## Conclusions

The occupational exposure of healthy subjects to nanoparticles increases the concentration of pro-inflammatory cytokines such as TNF-α. In our study, the most harmful effect of the nanoparticles on the immune system as an inflammation was found among the wood processing workers (elevated levels of TNF-α in the serum and in the nasal lavage, and a much higher concentration of IL-8 in the nasal lavage at the end of the working week).

The results of SEM analysis show that dust particles from the workplaces comprise all three size groups of particles (microscopic, ultramicroscopic and nanometric particles containing inorganic and organic dust) and that particles from the metal industry samples contain more dust of ultramicroscopic and nanometric size and less dust of microscopic size (both inorganic and organic). Samples from the woodworking industry contained more microscopic and organic dust. We suggest the effect of the workplace nanoparticles can cause inflammatory reactions in organisms and could cause a predisposition for the development of possible inflammation-associated diseases in the future.

### Limitations of the study

According to researchers’ experience during this study, several limitations were highlighted for the interpretation of the findings to whole worker cohorts of the tested industries. Occupational data consisted of only of three enterprises from the typical industries, and health-related data were also limited to the number of healthy subjects (only 36). The appropriate equipment for personal sampling of occupational nanoparticle exposure is not still developed; therefore, air sampling was performed at the distance of 1.5 m from the breathing area.
